# CCAAT/Enhancer-binding protein delta mediates glioma stem-like cell enrichment and ATP-binding cassette transporter ABCA1 activation for temozolomide resistance in glioblastoma

**DOI:** 10.1038/s41420-020-00399-4

**Published:** 2021-01-12

**Authors:** Shao-Ming Wang, Wen-Chi Lin, Hong-Yi Lin, Yen-Lin Chen, Chiung-Yuan Ko, Ju-Ming Wang

**Affiliations:** 1 Cellular Pathobiology Section, Integrative Neuroscience Research Branch, Intramural Research Program, National Institute on Drug Abuse, NIH/DHHS, Baltimore, MD 21224 USA; 2 Graduate Institute of Neural Regenerative Medicine, College of Medical Science and Technology, Taipei Medical University, Taipei, Taiwan; 3 Department of Biotechnology and Bioindustry Sciences, College of Bioscience and Biotechnology, National Cheng Kung University, Tainan, Taiwan; 4 Ph.D. Program for Neural Regenerative Medicine, College of Medical Science and Technology, Taipei Medical University and National Health Research Institutes, Zhunan, Taiwan; 5 Department of Pathology, Cardinal Tien Hospital, School of Medicine, College of Medicine, Fu Jen Catholic University, New Taipei City, Taiwan; 6 TMU Research Center of Neuroscience, Taipei Medical University, Taipei, Taiwan; 7 TMU Research Center of Cancer Translational Medicine, Taipei, Taiwan; 8 Graduate Institute of Medical Sciences, College of Medicine, Taipei Medical University, Taipei, Taiwan; 9 International Research Center for Wound Repair and Regeneration, National Cheng Kung University, Tainan, Taiwan; 10 Graduate Institute of Medicine, College of Medicine, Kaohsiung Medical University, Kaohsiung, Taiwan

**Keywords:** CNS cancer, Diseases of the nervous system

## Abstract

Glioblastoma (GBM) is the most aggressive brain tumor and relapses after chemo- or radiotherapy in a short time. The anticancer drug temozolamide (TMZ) is commonly used for GBM treatment, but glioma stem-like cells (GSCs) often lead to drug resistance and therapeutic failure. To date, the mechanism of GSC formation in TMZ-treated GBM remains largely unknown. CCAAT/Enhancer-binding protein delta (CEBPD) is an inflammation-responsive transcription factor and is proposed to be oncogenic in the context of drug resistance, prompting us to clarify its role in TMZ-resistant GBM. In this study, we first found that the CEBPD protein levels in GBM patients were significantly increased and further contributed to TMZ resistance by promoting GSC formation. Accordingly, the protein levels of stemness transcription factors, namely, SRY-box transcription factor 2 (SOX2), octamer-binding transcription factor 4 (OCT4), NANOG, and ATP-binding cassette subfamily A member 1 (ABCA1), were increased in GSCs and TMZ-treated GBM cells. Increased binding of CEBPD to promoter regions was observed in GSCs, indicating the direct regulation of these GSC-related genes by CEBPD. In addition, an ABCA1 inhibitor increased the caspase 3/7 activity of TMZ-treated GSCs, suggesting that TMZ efflux is controlled by ABCA1 activity and that the expression levels of the ABCA1 gene are an indicator of the efficiency of TMZ treatment. Together, we revealed the mechanism of CEBPD-mediated GSC drug resistance and proposed ABCA1 inhibition as a potential strategy for the treatment of TMZ-resistant GBM.

## Introduction

Glioblastoma (GBM) is a type of brain tumor that arises from astrocytes^1^. There is a consensus, however, that the current treatments for GBM are ineffective. Temozolomide (TMZ) is used as a first-line therapy for GBM treatment due to its DNA-damaging effect^2,3^. TMZ, a small lipophilic molecule, is an orally available monofunctional DNA alkylating agent that crosses the blood–brain barrier (BBB)^2^. Evidence indicates that cancer stem cells, especially GBM stem cells (GSCs), are generally characterized as a population of cells within the tumor bulk that participate in self-renewal, tumor initiation, and drug resistance^4,5^. Therefore, GSCs play an important role in drug resistance in TMZ-treated glioma^6,7^. However, the mechanisms of GSC formation and drug resistance in TMZ-treated glioma remain largely unclear.

CCAAT/enhancer-binding protein delta (CEBPD), an inflammation-responsive transcription factor, has been recognized as an essential player in inflammatory disease and cancer progression^8–12^. Previous studies have shown that CEBPD is involved in cell anti-apoptosis processes^13^, cell migration^9^, reactive oxygen species (ROS) formation^10^, and cancer stemness^12^. Recent evidence demonstrates a critical role for CEBPD in glioma stemness due to PDGFA expression in response to inflammatory cytokine treatment^12^. However, the exact mechanism that links CEBPD to the genesis or processes of GSCs remains largely unknown. In particular, the role of CEBPD in the anti-apoptosis mechanisms of TMZ-resistant GSCs is unclear.

GSCs are involved in stemness maintenance and drug resistance due to their expression of stem cell transcription factors. Among these transcription factors, sex-determining region Y-Box (SOX2), octamer-binding transcription factor 4 (OCT4), and Nanog homeobox (NANOG) are critical components for maintaining pluripotency in embryonic stem cells (ESCs) and somatic stem cells^14^. SOX2, OCT4, and NANOG are known to be highly expressed in subpopulations of GSCs, maintaining self-renewal and cellular proliferation^15–17^.

Another key protein is ATP-binding cassette subfamily A member 1 (ABCA1), which is one of the ABC transporter membrane proteins that is expressed in GSCs and contributes to the movement of a wide variety of materials, such as drugs, lipids, and metabolic products, across the plasma and intracellular membranes^18,19^. Nonetheless, the molecular mechanism of action of stem cell transcription factors and ABC transporters in TMZ-treated glioma remains a critical question. In this manuscript, we focused on stem cell transcription factors and ABC transporters in TMZ-treated glioma to establish whether CEBPD affects cancer stemness and drug resistance in TMZ-treated glioma.

In this paper, we used a bioinformatic dataset to analyze relative gene expression in glioma patients, as well as caspase 3/7 activity and spheroid assays and molecular biology techniques to examine cell death, stemness features, and gene regulation. We found that CEBPD is related to the increase in glioma stemness and TMZ resistance in glioma cells. Stem cell transcription factors (SOX2, OCT4, and NANOG) and ABCA1 are responsive to regulation by CEBPD, which directly binds to the promoter regions of those genes in glioma spheroid cells and TMZ-treated glioma cells. Taken together, our results suggest CEBPD as a therapeutic target to block certain actions of TMZ-resistant glioma and glioma stemness.

## Results

### CEBPD is expressed at high levels in GBM patients and correlates with poor survival probability

**Fig. 1:**
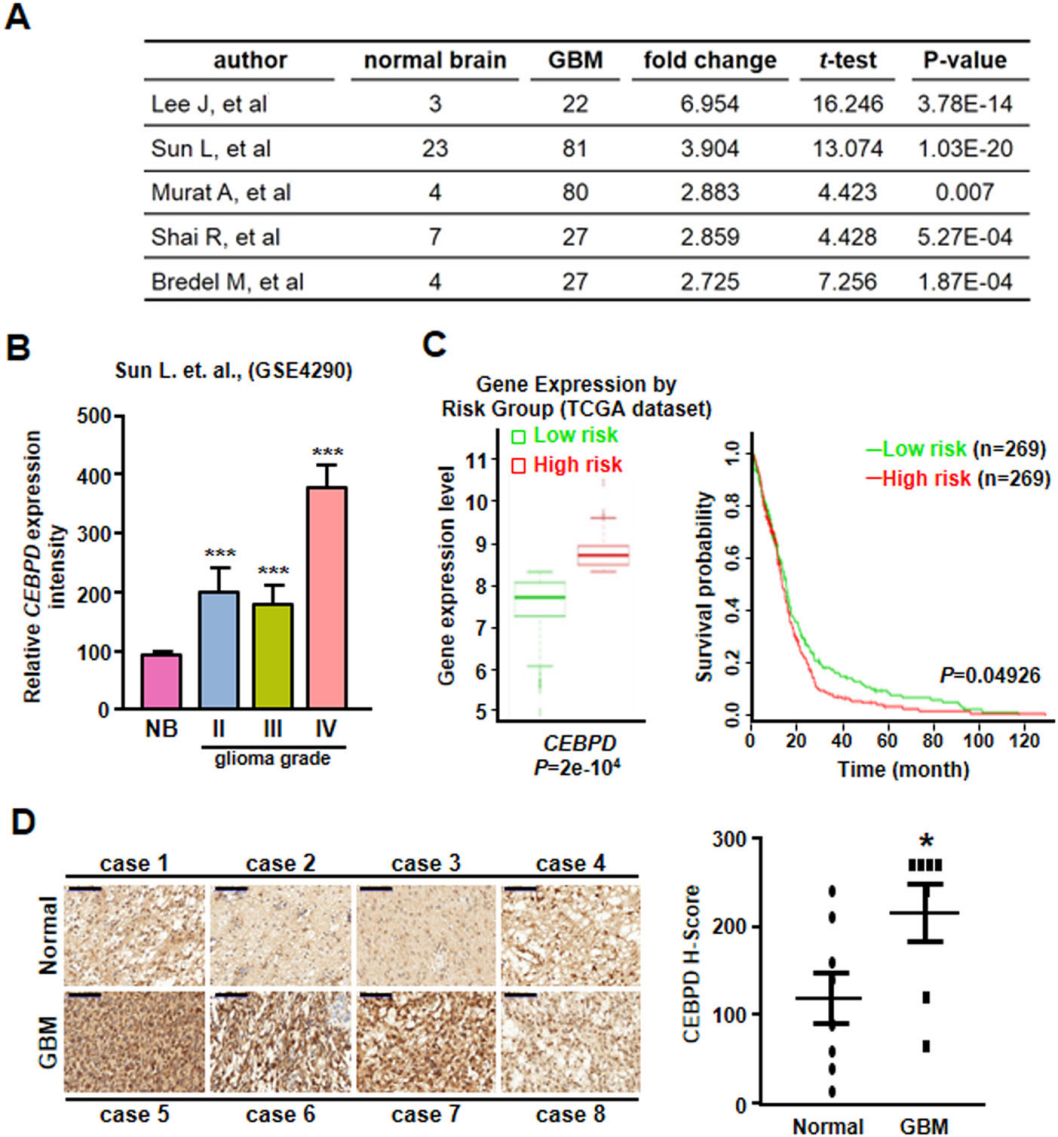
Relative CEBPD expression is higher in GBM and is associated with a poor survival rate. **A** GEO database analyses showing higher CEBPD expression in GBM tissues than in normal brain tissues. **B** CEBPD expression is higher in various grades of human glioma tissues. **C** Analysis of Kaplan–Meier survival curves of high-risk GBM patients and low-risk GBM patients from TCGA. **D** Representative immunohistochemistry staining of CEBPD in GBM and normal brain tissues. The CEBPD H-score value is higher in GBM samples than in normal samples. Scale bar, 100 μm. The summary data are presented as the mean ± SEM; the numbers in the bars represent the sample sizes; one-way ANOVA and Student’s *t*-test; *p < 0.05, ***p < 0.001.

Glioma GEO datasets showed that *CEBPD* messenger RNA (mRNA) levels are higher in GBM tissues than in normal brain tissues (Fig. 1A, B). Importantly, CEBPD significantly correlated with a poor prognosis of GBM (Fig. 1C). The immunohistochemical results showed that CEBPD levels are increased in GBM tissues compared with normal brain tissues (Fig. 1D).

### CEBPD is upregulated in GSCs and contributes to TMZ resistance

**Fig. 2:**
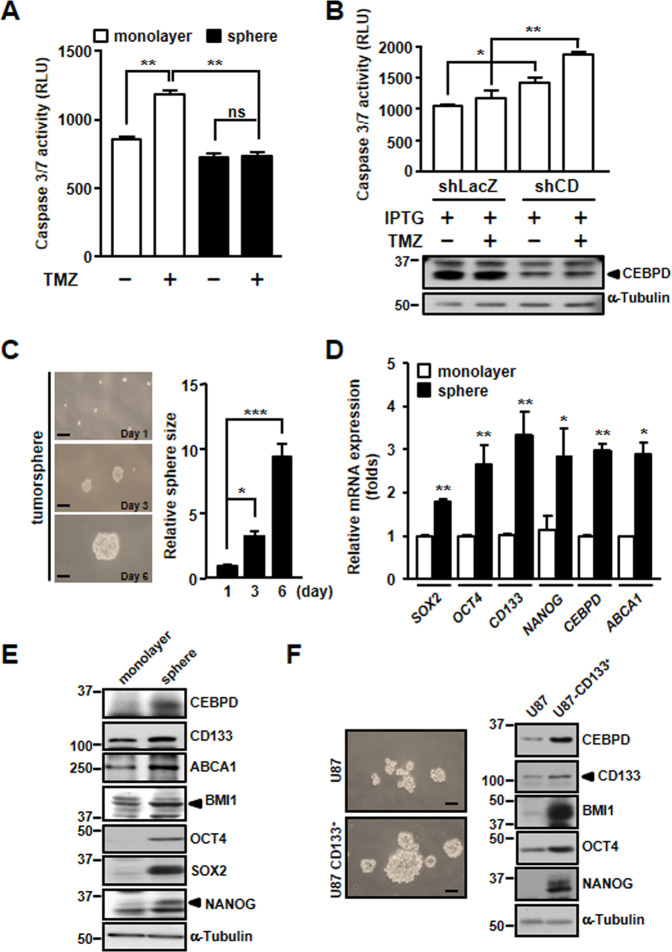
CEBPD is involved in intrinsic TMZ resistance of GSCs. **A** Caspase 3/7 activity is decreased in TMZ-treated GSCs. U87MG cells were cultured in stem cell medium for 3 days to form spheroids and treated with 50 μM TMZ for 72 h. The caspase 3/7 activity was detected by the Caspase-Glo^®^ 3/7 Reagent. **B** Attenuated CEBPD increases caspase 3/7 activity in TMZ-treated GSCs. Knockdown of CEBPD was conducted in U87MG stable cells with IPTG for 48 h, and then, the cells were cultured in stem cell medium for 3 days to form spheroids and treated with 50 μM TMZ for 72 h. The caspase 3/7 activity was determined by the Caspase-Glo^®^ 3/7 Reagent. **C** U87MG cells were cultured in stem cell medium to form spheroids, and the spheroid size was increased at 3 and 6 days. **D** RT-qPCR analyses and (**E**) western blots were performed with total RNA and total cell lysates harvested from U87MG monolayer cells and U87MG-derived spheroids. **F** U87MG-CD133^+^ cells express high levels of CEBPD and stemness-related proteins. U87MG and U87MG-CD133^+^ cells were cultured in stem cell medium for 3 days to form spheroids and cell lysates were harvested for western blot analysis. Scale bar, 100 μm. The summary data are presented as the mean ± SEM; the numbers in the bars represent the sample sizes; one-way ANOVA and Student’s *t*-test; *p < 0.05, **p < 0.01, ***p < 0.001, ns: no significant, shLacZ: shβ-galactosidase, shCD: shCEBPD.

A previous study showed that GSCs are more resistant to chemo- and radiotherapy and further contribute to GBM recurrence^20^. To evaluate this phenomenon in our system, we cultured U87MG-derived spheroids to enrich GSCs since a spheroid environment can be applied to enrich cancer stem cells (CSCs)^21,22^ and then treated them with TMZ. As expected, caspase 3/7 activity was decreased in GSCs compared with monolayer GBM cells treated with TMZ (Fig. 2A). Notably, knockdown of CEBPD in GSCs led to increased cell death (Fig. 2B). These results suggested that CEBPD mediates TMZ resistance in GSCs.

To further examine the role of CEBPD in GSCs, a spheroid-forming assay was conducted. We found that the spheroid size of U87MG cells gradually increased on different days in GSC-enriched culture medium (Fig. 2C). Furthermore, the expression levels of stemness factors (CD133, SOX2, OCT4, and NANOG) and CEBPD were elevated in GSCs (Fig. 2D, E). We further sorted GSCs from U87MG cells based on the CD133 marker and found that the expression of CEBPD was higher in U87MG-CD133^+^ cells (Fig. 2F).

### CEBPD regulates stemness-related genes

**Fig. 3:**
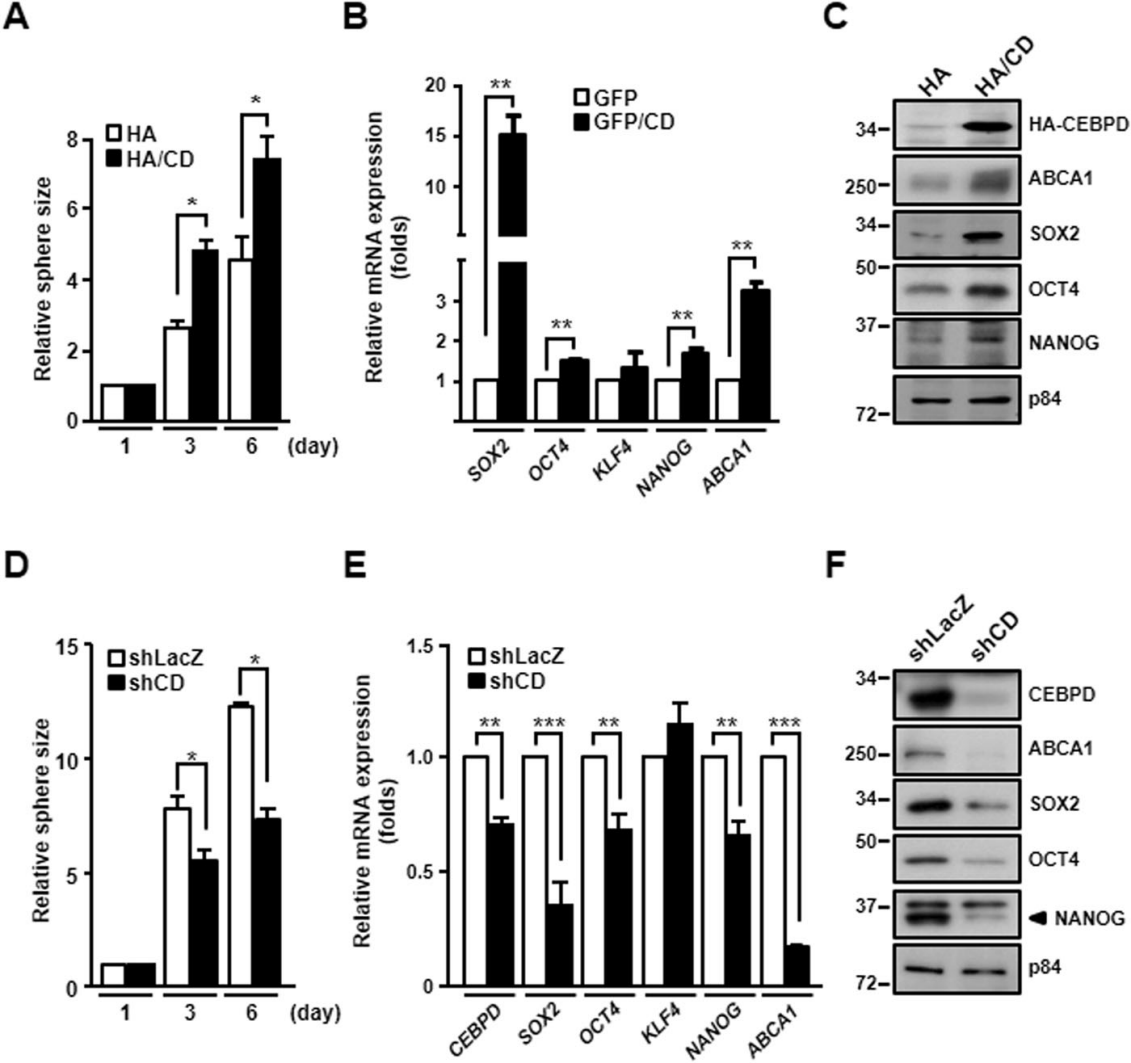
CEBPD promotes spheroid formation by regulating stemness-related factors and ABCA1 expression. **A** U87MG cells transiently expressing HA or HA-CEBPD were cultured in stem cell medium to form spheroids for 3 days and 6 days. **B** U87MG cells transiently expressing GFP or GFP-CEBPD were cultured in stem cell medium to form spheroids for 6 days. Total RNA were harvested and examined by RT-qPCR to detect the expression of *SOX2*, *OCT4*, *NANOG*, *KLF4*, and *ABCA1*. **C** Total protein lysates were harvested from (**A**) and examined by western blot to detect the expression of SOX2, OCT4, NANOG, KLF4, and ABCA1. **D** LacZ-knockdown or CEBPD-knockdown U87MG cells were cultured in stem cell medium to form spheroids for 3 days and 6 days. **E** Total RNA and **F** total protein lysates were harvested from shLacZ-U87MG or shCD-U87MG spheroid cells and examined by RT-qPCR and western blot to detect the transcription and expression of SOX2, OCT4, NANOG, KLF4, and ABCA1. The summary data are presented as the mean ± SEM; the numbers in the bars represent the sample sizes; one-way ANOVA and Student’s *t*-test; *p < 0.05, **p < 0.01, ***p < 0.001. HA: hemagglutinin, HA/CD: HA-tagged CEBPD, GFP: green fluorescent protein, GFP/CD: GFP-tagged CEBPD.

Stemness-related factors OCT4, SOX2, NANOG, and KLF4 are common transcriptional regulators in cancer stem cells^23^. In particular, SOX2, OCT4, and NANOG can maintain the self-renewal and stemness properties of GSCs^15–17^. To elucidate whether CEBPD regulates these factors, gain- and loss-of-function experiments were conducted. The spheroid size was increased in CEBPD-overexpressing GSCs compared with control GSCs (Fig. 3A). The expression levels of SOX2, OCT4, and NANOG were upregulated in CEBPD-overexpressing GSCs (Fig. 3B, C). However, the spheroid size was decreased in CEBPD-knockdown GSCs (Fig. 3D). The gene and protein expression levels of SOX2, OCT4, and NANOG were downregulated in CEBPD-knockdown GSCs (Fig. 3E, F). These results suggested that CEBPD expression was related to the stem-like properties and drug resistance of GSCs by regulating SOX2, OCT4, NANOG, and ABCA1 transcription and expression.

### ABCA1 positively correlates with CEBPD expression and is involved in TMZ resistance

**Fig. 4:**
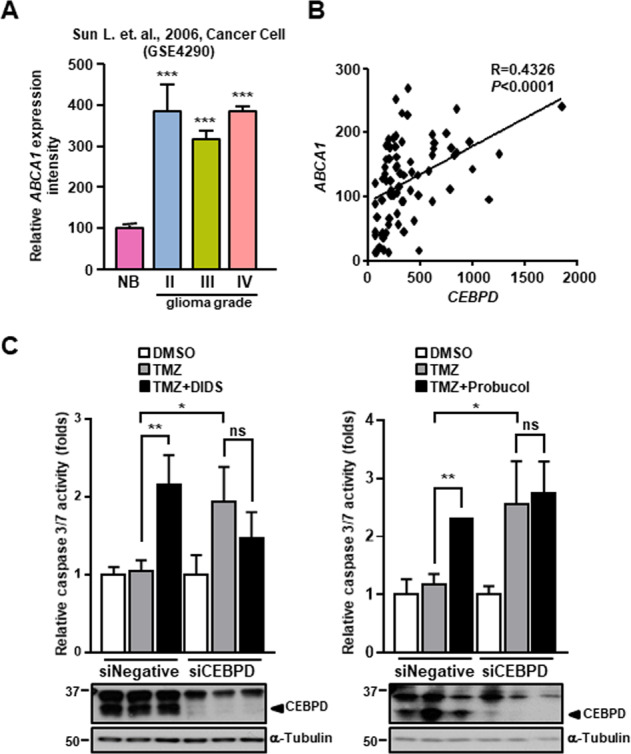
ABCA1 expression is correlated with CEBPD expression and promotes against TMZ-induced cell death in GSCs. **A** The relative *ABCA1* expressions are higher in various grades of human glioma tissues. Expression of *ABCA1* was analyzed in tumor tissues of glioma patients compared with normal tissues from the GEO database. **B** The mRNA expression of *ABCA1* positively correlates to *CEBPD* in glioma samples. **C** The CEBPD-ABCA1 axis mediates TMZ resistance in GSCs. The knockdown control (siNegative) or CEBPD-knockdown (siCEBPD) U87MG cells were cultured in stem cell medium for 2 days to form spheroids, pretreated with ABCA1 antagonists (400 μM DIDS and 10 μM Probucol) for 24 h, and then treated with 50 μM TMZ for 72 h. The caspase 3/7 activity was detected by the Caspase-Glo^®^ 3/7 Reagent. The summary data are presented as the mean ± SEM; the numbers in the bars represent the sample sizes; one-way ANOVA; *p < 0.05, **p < 0.01, ***p < 0.001, ns: no significant.

Multidrug resistance is a serious problem that hinders the success of cancer pharmacotherapy. A common mechanism of multidrug resistance is the overexpression of ABC efflux transporters in cancer cells^18,24^. According to our previous microarray data, ABCA1 is upregulated in response to CEBPD activation in U373MG cells^25^. The GEO dataset of glioma showed that the *ABCA1* level was higher in GBM tissues than in normal brain tissues (Fig. 4A). Moreover, the ABCA1 expression level was highly corresponded with the CEBPD expression level in these samples (Fig. 4B). The expression level of ABCA1 is also elevated in GSCs and regulated by CEBPD (Figs. 2D, E and 3B, C, E, F). To further elucidate whether ABCA1 is involved in TMZ resistance, a caspase 3/7 activity assay was conducted following treatment with ABCA1 antagonists, DIDS or Probucol, and TMZ. The results showed that caspase 3/7 activity was increased in GSCs following co-treatment with TMZ and the ABCA1 antagonists compared with TMZ. Furthermore, the cytotoxic effects of ABCA1 antagonists were eliminated in CEBPD-knockdown spheroids (Fig. 4C). These results suggested that ABCA1 participates in CEBPD-mediated TMZ resistance in GSCs.

### *SOX2*, *OCT4*, *NANOG,* and *ABCA1* genes are direct targets of CEBPD

**Fig. 5:**
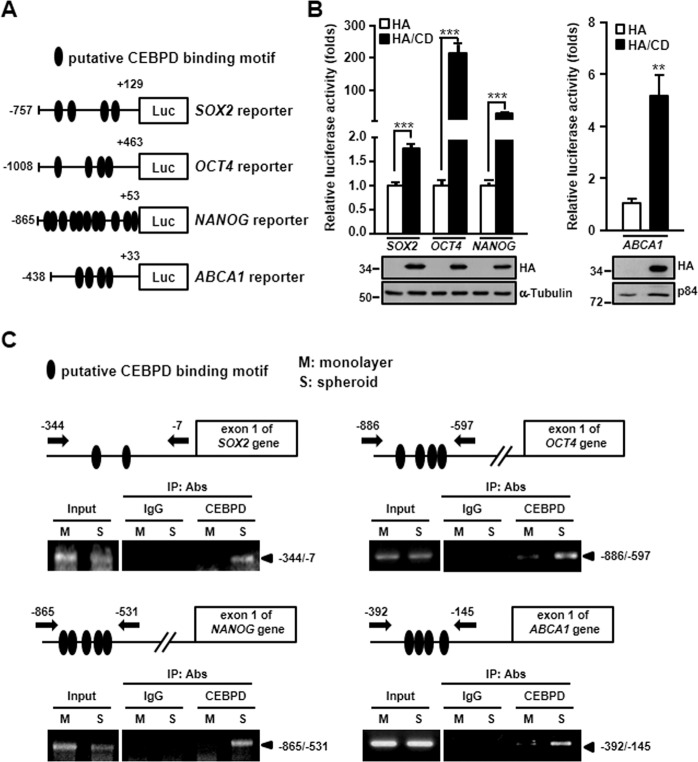
CEBPD can directly bind to the SOX2, OCT4, NANOG, and ABCA1 promoter regions to regulate gene expression. **A** Schematic representation of reporter constructs with the *SOX2*, *OCT4*, *NANOG*, and *ABCA1* promoters. The approximate locations of the putative CEBPD-binding motifs are indicated by ovals. **B** U87MG cells were co-transfected with pcDNA3-HA or pcDNA3-HA/CEBPD and SOX2, OCT4, NANOG, and ABCA1 reporter vectors and then examined by reporter assay. **C** CEBPD binds to the *SOX2*, *OCT4*, *NANOG,* and *ABCA1* promoters in vivo. Chromatins from U87MG monolayer or spheroid cells were isolated, and ChIP assays were performed with the indicated antibodies. The precipitated DNA was amplified by PCR using specific primers. The summary data are presented as the mean ± SEM; the numbers in the bars represent the sample sizes; Student’s *t*-test; **p < 0.01, ***p < 0.001.

As CEBPD is a transcription factor, we next examined whether *SOX2*, *OCT4*, *NANOG*, and *ABCA1* are downstream targets of CEBPD. According to the prediction website for transcription factor binding (http://alggen.lsi.upc.es/cgi-bin/promo_v3/promo/promoinit.cgi?dirDB=TF_8.3), several CEBPD binding sites were identified in the *SOX2*, *OCT4*, *NANOG*, and *ABCA1* promoter regions (Fig. 5A). The promoter activities of *SOX2*, *OCT4*, *NANOG*, and *ABCA1* were upregulated in CEBPD-overexpressing cells (Fig. 5B). Furthermore, an in vivo DNA binding assay showed that CEBPD bound to the *SOX2*, *OCT4*, *NANOG*, and *ABCA1* promoter regions in U87MG-derived spheroids (Fig. 5C). These results suggested that CEBPD can upregulate SOX2, OCT4, NANOG, and ABCA1 expression in GSCs.

### CEBPD mediates the activation of stemness-related factors and ABCA1 in TMZ-treated GBM cells

**Fig. 6:**
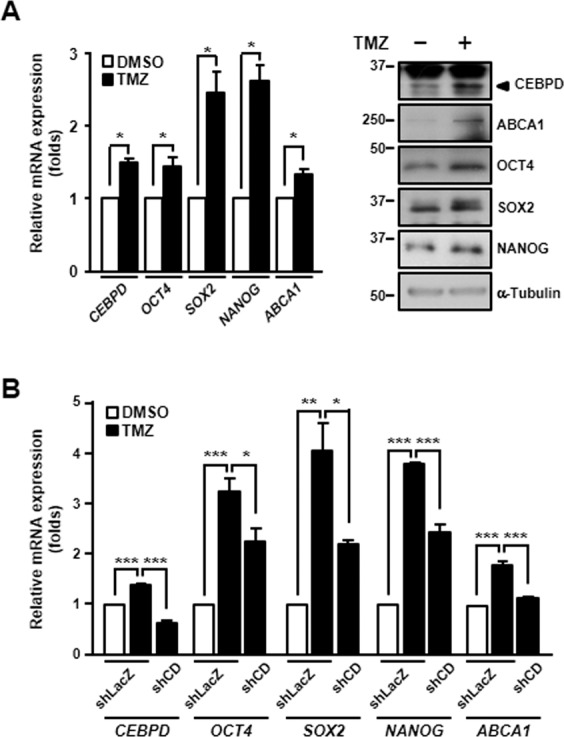
CEBPD mediates *SOX2*, *OCT4*, *NANOG*, and *ABCA1* upregulation in TMZ-treated GBM cells. **A** The expression of CEBPD, SOX2, OCT4, NANOG, and ABCA1 is increased in TMZ-treated U87MG cells. Monolayer U87MG cells were treated with 50 μM TMZ for 6 h (for RNA) or 24 h (for protein). Total RNA and total protein lysates were harvested, and then, the SOX2, OCT4, NANOG, and ABCA1 transcription and expression levels were assessed by RT-qPCR and western blot. **B** Knockdown of CEBPD downregulates *SOX2*, *OCT4*, *NANOG*, and *ABCA1* transcripts in TMZ-treated GBM cells. Knockdown of CEBPD was conducted in U87MG stable cells with IPTG for 48 h, and then, the cells were treated with 50 μM TMZ for 6 h. Total RNA was harvested, and the *CEBPD*, *SOX2*, *OCT4*, *NANOG*, and *ABCA1* expression levels were assessed by RT-qPCR. The summary data are presented as the mean ± SEM; the numbers in the bars represent the sample sizes; one-way ANOVA and Student’s *t*-test; *p < 0.05, **p < 0.01, ***p < 0.001.

A previous study showed the phenotypic shift in the non-GSC population to a GSC-like state in GBM after primary chemotherapy^26^. To examine whether CEBPD is involved in acquired TMZ resistance in GBM by inducing stemness, we treated U87MG monolayer cells with TMZ and found that CEBPD was induced (Fig. 6A). Moreover, SOX2, OCT4, NANOG, and ABCA1 were also activated in TMZ-treated cells (Fig. 6A). These results suggested that TMZ could contribute to the activation of stemness and acquisition of TMZ resistance. As CEBPD was responsive to TMZ, we further examined whether CEBPD is involved in TMZ-induced stemness and acquired TMZ resistance. In CEBPD-knockdown U87MG cells, the *SOX2*, *OCT4, NANOG*, and *ABCA1* transcripts were downregulated following TMZ treatment (Fig. 6B). These results showed that CEBPD contributed to TMZ-induced *SOX2*, *OCT4*, *NANOG*, and *ABCA1* activation. In accordance with the results from spheroid cells and TMZ-treated GBM cells, we showed that CEBPD serves as a key regulator of TMZ resistance in GBM by regulating SOX2, OCT4, NANOG, and ABCA1 at the molecular and transcriptional levels.

## Discussion

CSCs contribute to tumor initiation, drug resistance, and tumor relapse and are associated with tumor progression^5,20^. While there is evidence that suggests that small subpopulations of CSCs exist in the tumor bulk, the exact molecular mechanism of CSC formation in the context of anticancer drug treatment remains largely unclear. Previous studies have shown that GBM leads to poor prognosis for patients due to the presence of glioma stem-like cells^27^. Inasmuch as the drug resistance properties of GSCs protect gliomas against cell death, leading to GBM progression, the molecular mechanisms of action of drug resistance and stemness properties in CSCs, particularly GSCs, remain a critical question.

CEBPD is a well-known transcriptional factor that is activated by inflammatory cytokines and anticancer drug treatment^11,12^. CEBPDs play a dual function in cancer biology. In liver cancer and leukemia, CEBPD plays a tumor suppressive role by inducing cell death^28,29^. In bladder cancer and glioma, CEBPD plays an oncogenic role by increasing drug resistance and cancer stemness^11,12^. Although no single mechanism controls the biological functions of cancer, we can elucidate the kinds of cancer in which CEBPD activation plays a tumor suppressive or oncogenic role. A previous study showed that CEBPD activation can regulate PDGFA transcription, which promotes GSC formation in response to inflammatory cytokine treatment^12^. Whether CEBPD can directly regulate stemness-related factors to form CSCs remains unknown.

Our results show that CEBPD can directly bind to the *SOX2*, *OCT4*, *NANOG*, and *ABCA1* promoter regions to promote the properties of cancer stemness and drug resistance. Interestingly, some studies suggest that ABCA1 is involved in drug resistance and upregulated in side population cells^24,30,31^. We also found that treatment with ABCA1 antagonists, such as DIDS and Probucol, can increase GSC death following TMZ treatment, suggesting that ABCA1 plays a key role in preventing cell death. TMZ treatment can increase ABCA1 transcription and expression through CEBPD activation. CEBPD could be activated by TMZ stimulation due to induction of stress. Previous studies showed that TMZ treatment can increase ROS imbalance^6^ and genotoxic stress^32^, suggesting that TMZ-induced CEBPD expression may promote drug resistance by stimulating ROS stress. However, how these upstream mechanisms affect CEBPD remains to be elucidated in the future.

The findings here show that CEBPD can regulate SOX2, OCT4, and NANOG expression. It will be interesting to determine whether SOX2, OCT4, and NANOG can directly regulate ABCA1 expression. ABCA1 may be regulated by two possible mechanisms: (a) stemness-related factors (SOX2, OCT4, and NANOG) may regulate ABCA1 expression through promoter regulation or microRNA regulation and (b) CEBPD may directly interact with stemness factors to bind to CEBPD, SOX2, OCT4, and NANOG binding sites in the ABCA1 promoter to regulate ABCA1 expression. The detailed mechanism needs to be further clarified.

**Fig. 7:**
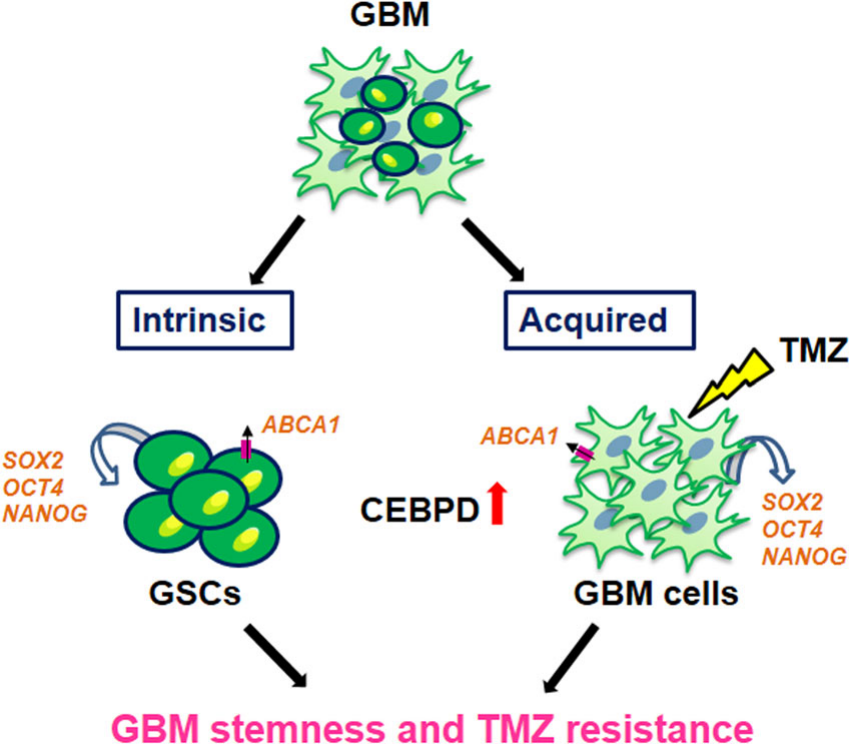
A proposed model for the involvement of CEBPD in stemness and TMZ resistance in GBM. CEBPD can facilitate GSC formation and GBM resistance to TMZ by upregulating the SOX2, OCT4, NANOG, and ABCA1 expression through acquired and intrinsic regulation.

In summary, we have found that this novel role of CEBPD may represent a clinical target for treating TMZ-resistant glioma. We have also found that CEBPD can facilitate GSC formation and drug resistance by binding to the SOX2, OCT4, NANOG, and ABCA1 promoter regions. ABCA1 antagonists may act as therapeutic agents to inhibit GSC-induced drug resistance (Fig. 7). Thus, targeting CEBPDs or ABCA1s may be a beneficial therapeutic approach, in combination with TMZ or anticancer drugs, to attenuate glioma progression.

## Materials and methods

### Materials

The TRIzol™ RNA extraction reagent, Lipofectamine® 2000 transfection reagent, Lipofectamine® RNAiMAX transfection reagent, Opti-MEM medium, Dulbecco’s modified Eagle’s medium (DMEM), B-27™ Supplement (50X) (17504044), and fetal bovine serum (FBS) were purchased from Thermo Fisher Scientific (Waltham, MA, USA). PrimeScript™ RT reagent kit was purchased from TaKaRa (Kusatsu, Shiga, Japan). SensiFAST™ SYBR was purchased from Bioline (Taunton, MA, USA). A luciferase assay system and the Caspase-Glo® 3/7 assay was purchased from Promega (Madison, WI). Human EGF (GFH26) was purchased from Cell Guidance Systems (Cambridge, UK) and Human FGF-basic (100-18B) was purchased from PeproTech (Rocky Hill, NJ, USA). 4,4′-Diisothiocyanatostilbene-2,2′-disulfonic acid disodium salt hydrate (DIDS) (D3514), Probucol, Temolozomide (SI-T2577), and antibody against α-Tubulin (T6199) were purchased from Sigma (St. Louis, MO, USA). An antibody against CEBPD (SC-636) and poly-(2-hydroxyethyl methacrylate) (sc-253284) were purchased from Santa Cruz Biotechnology (Santa Cruz, CA, USA). Antibodies against p84 (GTX70220), SOX2 (GTX101507), NANOG (GTX100863), and ABCA1 (GTX27360) were purchased from GeneTex (Irvine, CA, USA). Antibodies against OCT4 (#2750) were purchased from Cell Signaling Technology (Danvers, MA, USA). An antibody against HA was purchased from Covance (Barkeley, CA). An antibody against CEBPD for immunostaining was purchased from Abcam (ab65081, Cambridge, MA, USA). All oligonucleotides were synthesized by PURIGO biotechnology (Taipei, Taiwan). Expression plasmid pcDNA3/HA and pEGFP-C1 were a gift of Dr. Hsin-Fang Yang-Yen (Institute of Molecular Biology, Academia Sinica, Tapei, Taiwan).

### Cell culture

Human GBM cell line U87MG was maintained in Dulbecco’s modified Eagle’s medium supplemented with 10% FBS, 100 μg/ml streptomycin, and 100 μg/ml penicillin. Inducible knockdown LacZ U87MG cells and inducible knockdown CEBPD U87MG cells were maintained in above medium with 500 μM IPTG.

### Spheroid formation assay

U87MG cells were seeded and cultured in DMEM/F12 medium. The medium was supplemented with B27, 20 ng/ml EGF and 10 ng/ml bFGF. The dished were coated with poly-(2-hydroxyethyl methacrylate). After 3 days and 6 days, the sizes of spheroids were measured at 100X magnification under a microscope and analyzed by ImageJ software.

### Reverse transcription polymerase chain reaction (RT-qPCR)

After cells were exposed to simulation, the total RNA was harvested and extracted in TRIzol^TM^. The isolated RNA was subjected to reverse transcription reaction with PrimeScript^TM^ for cDNA synthesis. The oligonucleotide primers used in the qPCR analysis were as follows: GAPDH specific primer (F): 5′-CCACCCAGAAGACTGTGGAT-3′ and GAPDH specific primer (R): 5′-TTCAGCTCAGGGATGACCTT-3′; human CEBPD specific primer (F): 5′-GCCATGTACGACGACGAGAG-3′ and CEBPD specific primer (R): 5′-TGTGATTGCTGTTGAAGAGGTC-3′; human CD133 specific primer (F): 5′-CCAAGTTCTACCTCATGTTTGG-3′and CD133 specific primer (R):5′-ACCAACAGGGAGATTGCAAAGC-3′; human SOX2 specific primer (F): 5′-CACAACTCGGAGATCAGCAA-3′ and SOX2 specific primer (R):5′- CTCCGGGAAGCGTGTACTTA-3′; human OCT4 specific primer (F): 5′-GGAAGGTATTCAGCCAAACGACCA-3′ and OCT4 specific primer (R):5′-CTCACTCGGTTCTCGATACTGGTT-3′; human NANOG specific primer (F): 5′-ACCAGAACTGTGTTCTCTTCCACC-3′ and NANOG specific primer (R):5′- CCATTGCTATTCTTCGGCCAGTTG-3′; human KLF4 specific primer (F): 5′-CCCAATTACCCATCCTTCCT-3′ and KLF4 specific primer (R):5′-AGGTTTCTCACCTGTGTGGG-3′;human ABCA1 specific primer (F): 5′-AACAGTTTGTGGCCCTTTTG-3′ and ABCA1 specific primer (R):5′-AAGTTCCAGGCTGGGGTACT-3′.

### Plasmid transfection

Cells were re-plated 24 h before transfection at an optimal density in 2 ml of fresh culture medium in a 6-well plastic dish. They were then transfected with plasmids by Lipofectamine^®^ 2000 transfection reagent according to the manufacturer’s instructions. The total amount of DNA for each experiment was matched with the empty vector. The Opti-MEM media were changed to culture medium after 6 h, incubated for 15 h and harvested for further analysis.

### Plasmid construction and reporter assay

The pcDNA3/HA/CEBPD (HA/CD) was constructed previously^33^. The CEBPD fragment was digested with BamHI and XbaI from HA/CD and inserted into BamHI- and XbaI-digested pEGFP-C1 to produce GFP-tagged CEBPD (GFP/CD). The 5′ promoter regions of *NANOG*, *OCT4*, *SOX2*, and *ABCA1* genes were obtained by PCR with U87MG genomic DNA. The PCR products were individually cloned into pGL-3 basic vector. The primers for the PCR of the genomic DNA were as follows: SOX2 (F): 5′-MluI-CGACGCGTCGGTTGGACAGGGAGATGGCAGCTTA-3′; SOX2 (R): 5′-BglII-GAAGATCTTCCTCTCCTCTTCTTTCTCTCAGTCC-3′; OCT4 (F): 5′-MluI-CGACGCGTCGACAGATCACTTGAGGTCTCCTGA-3′; OCT4 (R): 5′-BglII-GAAGATCTTCTCACTCAAGTATCACCCCCAGTT-3′; NANOG (F): 5′-MluI-CGACGCGTCGCTGGGAGGAGGGATAGACAAGAAA-3′; NANOG (R): 5′-BglII-GAAGATCTTCCAGCTCAGTCCAGCAGAACGTTAA-3′; ABCA1(F)-(−438): 5′-KpnI–GGTACCGAAAGGAAACAAAAGACAAG-3′; ABCA1 (R)-(+33): 5′-HindIII-AAGCTTACTATCGGTCAAAGCCTGTG3′. After transfection, the luciferase activities in cell lysates were measured following the manufacturer’s instructions for the luciferase assay.

### Lentiviral short hairpin RNA (shRNA) assay

The virus was produced from Phoenix Ampho cells co-transfected with pMD2.G and psPAX2 vectors and the pLKO.1-shRNA expression vectors using Mirus Bio TransIT-X2. The shRNA targeting LacZ or CEBPD were sub-cloned into the lentiviral expression vector pLKO.1. The short hairpin RNA sequences are as follows: shLacZ: 5′-CCGGTGTTCGCATTATCCGAACCATCTCGAGATGGTTCGGATAATGCGAACATTTTTG-3′; shCEBPD (shCD): 5′-CCG GGCCGACCTCTTCAACAGCAATCTCGAGATTGCTGTTGAAGAGGTCGGCTTTTT-3′. The expression plasmids and shRNAs were purchased from the National RNAi Core Facility located at the Genomic Research Center of Institute of Molecular Biology, Academia Sinica, Taiwan.

### Small interfering RNA (siRNA) assay

The sequence of CEBPD knockdown siRNA is CGACCUCUUCAACAGCAAUTT and the negative control siRNA sequence is not found in the human genome databases (Thermo Fisher Scientific, Waltham, MA, USA). Cells were transfected separately with CEBPD siRNA or negative control siRNA by Lipofectamine® RNAiMAX transfection reagent according to the manufacturer’s instructions. After 48 h, cells were harvested for further analysis.

### Caspase 3/7 activity assay

The conditional media were prepared from different stimulations of cells and then equivalent amounts of Caspase-Glo^®^ 3/7 reagent were added to the 96-well plate. The samples were mixed on a shaker at room temperature for 30 min and the luciferase activity was measured with a luminometer.

### Western blotting assay

This assay was carried out as described previously^34^. Briefly, cells were lysed in modified radioimmune precipitation assay buffer. Following lysis, the lysates were resolved on an SDS polyacrylamide gel, transferred to a polyvinylidene difluoride membrane by an electroblot apparatus. Membranes were incubated with primary antibodies overnight at 4 °C and secondary antibodies at RT for 1 h. Proteins were detected by an enhanced chemiluminescence western blot system from Pierce (Rockford, IL, USA) and visualized by an autoradiographic film.

### Chromatin immunoprecipitation (ChIP) assay

The ChIP assay was carried out as described previously^34^. The cross-linked protein/DNA lysates were immunoprecipitated with specific antibodies recognizing CEBPD, or control rabbit immunoglobulin G (IgG) at 4 °C for 12–16 h. After reversal of the crosslinking between proteins and genomic DNA, the precipitated DNA was amplified by PCR with primers related to the specific regions on the genomic locus of target genes. The primers included SOX2 (F): 5′-GTACCCTGCACCAAAAAGT-3′; SOX2 (R): 5′-AGACAACCAT CCATGTGAC-3′; OCT4 (F): 5′-GAGGCAGAATAGCTTGAACC-3′; OCT4 (R): 5′-TGGAGGAGGGTGACACTTTT-3′; NANOG (F): 5′-CCTCAGGAATTTAAGGTGCATG-3′; NANOG (R): 5′-CCACCCCTGTAATCCCAGTAAA-3′; ABCA1 (F): 5′-ATTCAGCCTAGAGCT CTCTCT-3′; ABCA1 (R): 5′-AGGGTACAGCAGGTGTCTTAG-3′.

### Gene expression omnibus (GEO) database

The GEO databases used in this study are Lee data set (GSE4536), Sun data set (GSE4290), Bredel data set (GSE2223), Murat data set (GSE7696), and Shai data set^35^. These databases were used to access gene expression levels in normal and glioma tissues.

### Immunohistochemistry (IHC) analysis

The tissue arrays were purchased from Biomax (GL481, Rockville, MD, USA). The staining was performed using a Ventana BenchMark XT automated stainer (Ventana, Tucson, AZ, USA) with CEBPD primary antibody.

### Statistical analysis

Results are shown as mean ± SEM. All statistical analysis was conducted using the Prism GraphPad software. Student’s *t*-test or one-way analysis of variance (ANOVA) followed by Tukey’s multiple comparison test was used. The correlation analysis was determined by the Pearson correlation test. All experiments were repeated three times. Statistically significant differences were indicated by ***p < 0.001, **p < 0.01, *p < 0.05.
